# DIA proteomics analysis through serum profiles reveals the significant proteins as candidate biomarkers in women with PCOS

**DOI:** 10.1186/s12920-021-00962-7

**Published:** 2021-05-09

**Authors:** Ying Yu, Panli Tan, Zhenchao Zhuang, Zhejiong Wang, Linchao Zhu, Ruyi Qiu, Huaxi Xu

**Affiliations:** 1 Institute of Laboratory Medicine, Jiangsu Key Laboratory of Laboratory Medicine, Jiangsu University, No. 301 Xuefu Road, Zhenjiang, Jiangsu 210013 People’s Republic of China; 2 Department of Laboratory Medicine, Chinese Medicine Hospital of Zhejiang, Hangzhou, Zhejiang 310006 People’s Republic of China

**Keywords:** Proteomics analysis, Polycystic ovary syndrome, Proteome changes, Biomarkers

## Abstract

**Background:**

The aim of this study was to apply proteomic methodology for the analysis of proteome changes in women with polycystic ovary syndrome (PCOS).

**Material and methods:**

All the participators including 31 PCOS patients and 31 healthy female as controls were recruited, the clinical characteristics data was recorded at the time of recruitment, the laboratory biochemical data was detected. Then, a data-independent acquisition (DIA)-based proteomics method was performed to compare the serum protein changes between PCOS patients and controls. In addition, Western blotting was used to validate the expression of identified proteomic biomarkers.

**Results:**

There were 80 proteins differentially expressed between PCOS patients and controls significantly, including 54 downregulated and 26 upregulated proteins. Gene ontology and Kyoto Encyclopedia of Genes and Genomes analysis showed that downregulated proteins were enriched in platelet degranulation, cell adhesion, cell activation, blood coagulation, hemostasis, defense response and inflammatory response terms; upregulated proteins were enriched in cofactor catabolic process, hydrogen peroxide catabolic process, antioxidant activity, cellular oxidant detoxification, cellular detoxification, antibiotic catabolic process and hydrogen peroxide metabolic process. Receiver operating characteristic curves analysis showed that the area under curve of Histone H4 (H4), Histone H2A (H2A), Trem-like transcript 1 protein (TLT-1) were all over than 0.9, indicated promising diagnosis values of these proteins. Western blotting results proved that the detected significant proteins, including H4, H2A, TLT-1, Peroxiredoxin-1, Band 3 anion transport protein were all differently expressed in PCOS and control groups significantly.

**Conclusion:**

These proteomic biomarkers provided the potentiality to help us understand PCOS better, but future studies comparing systemic expression and exact role of these candidate biomarkers in PCOS are essential for confirmation of this hypothesis.

## Introduction

Polycystic ovary syndrome (PCOS) is one of the most common endocrine and metabolic disorders, 5–15% female in reproductive age live with a PCOS diagnosis worldwide. The etiopathogenesis of PCOS is complex, genetic, environmental and lifestyle interaction contribute to the etiology of PCOS. Hyperandrogenism and insulin resistance (IR) are the major characteristics of PCOS [1]. And normally, except for the above-mentioned characteristics, the clinical features of PCOS usually including menstrual cycle irregular, ovarian abnormalities, follicular dysplasia (with multiple cystic ovarian follicles), etc. [2]. But the diagnosis of PCOS is quite difficult since the symptoms of PCOS patients are not unified. Except for the PCOS caused physical hazards in reproductive age women, PCOS also has negative effects on life quality and mental health. In the female with PCOS, the long-term risks of endometrial carcinoma, diabetes mellitus, and cardiovascular diseases also seem to be higher than the normal populations [3, 4]. Population based studies show that PCOS and thereby caused infertility are associated with lower life satisfaction, poor health status and increased psychological distress, including depression, anxiety and perceived stress [5, 6].

Proteomics is an emerging tool involves comprehensive study of qualitative and quantitative profiling of proteins present in tested samples [7]. It can systematically characterize a large-scale of dynamic changes in protein expression, which can provide basic information for the study of complex diseases. Thus allowing the capacity to unravel new mechanistic explanations and offering richer source of potential diagnostic biomarkers associated with complex metabolic disorders. In the past few years, substantial efforts have been used to study the pathogenesis of PCOS via proteomic approaches, but challenges still exist [8]. The identification of novel proteins in PCOS is of great interest for developing more precise diagnostic strategy and new therapeutic targets. Data independent acquisition (DIA) recommended with high reproducibility and high-throughput is a powerful technique in proteomics studies. The mass spectrometer in DIA systematically acquires MS/MS spectra without regard to whether a precursor signal is detected, which is in comparison to datadependent acquisition (DDA) for proteomics research [9].

In this experiment, DIA proteomic techniques was employed to explore a broad spectrum of functional proteins in PCOS patients and controls. The aim of this study was to use proteomic methodologies for the identification of biomarkers over- or under expressed in women with PCOS compared with the controls, and provide the potentiality to help us understand PCOS better.

## Material and methods

### Regents

UltraPure™ Tris Hydrochloride (Tris–HCl) (Invitrogen, CA, USA), Ammonium biocarbonate (NH_4_HCO_3_) (Sigma-Aldrich, Shanghai, China), Trifluoroacetic Acid (TFA) (Sigma-Aldrich, Shanghai, China), DT-Dithiothreitol (DTT) (Sigma-Aldrich, Shanghai, China), Iodoacetamide (IAM) (Sigma-Aldrich, Shanghai, China), Lysyl Endopeptidase, MS Grade (LysC) (Wako, Japan), Sequencing Grade Modified Trypsin (Trypsin) (Promega, WI, USA), SOLAμ HRP 2 mg/1 mL 96 well plate (Thermo Scientific, Shanghai, China), Methanol, Optima ™ LC/MS Grade (Fisher Scientific, PA, USA), Acetonitrile, Optima™ LC/MS Grade, (Fisher Scientific, PA, USA), ddH_2_O (water from thermo ScientificTM water Purification system), High Select™ Top14 Abundant Protein Depletion Mini Spin Columns (Fisher Scientific, PA, USA).

### Study subjects

All the participators including 31 PCOS patients and 31 paired healthy female controls were recruited from the Chinese Medicine Hospital of Zhejiang province (Hangzhou, China). This study received the ethical approval from the Ethics Committee of Zhejiang Provincial Hospital of Chinese Medicine [approve no. 2020-KL-155-02]. All the participators signed informed consent forms before the start of the study.

The inclusion criteria for PCOS cases were: adolescent females, diagnosed with PCOS, had at least 2 years of menstrual history. And in order to exclude the luteal phase, progesterone (P) level was less than 3.82. PCOS was diagnosed based on the recent androgen excess and Rotterdam criteria, 2003: clinical or biochemical hyperandrogenism and ovarian dysfunction: oligomenorrhea (menstrual cycle of more than 45 days) and/or polycystic ovaries on ultrasound (ovarian volume > 10 mL in at least one ovary) [10]. Exclusion criteria: other disorders with similar presentation (hyperprolactinemia, thyroid disorders, late-onset congenital adrenal hyperplasia, androgen-secreting ovarian or adrenal tumors, Cushing syndrome, or other related disorders) were excluded [11]. Healthy controls were volunteers with age and gender matched with PCOS patients. And no evident disease was detected in them during the course of the study.

The clinical characteristics data of the enrolled participators were recorded at the time of recruitment. After fasting for 8 h, a venous blood sample from each participator was collected. The serum samples were stored at -80 ℃ for subsequent assay.

### Clinical laboratory tests

Serum concentrations of follicle-stimulating hormone (FSH), luteinizing hormone (LH), estradiol (E2), prolactin (PRL), testosterone (T), P in all PCOS patients and control participators were detected by Immulite 2000 analyzer (Siemens Healthcare Diagnostics Products Ltd., UK) using two site chemiluminescent immunometric assays.

### Clinical data analysis

All the clinical data were computed using SPSS18.0 version software. An unpaired, two-tailed student *t* test was performed on clinical biochemical data, the chi-square test was used for comparison of categorical variables. *p* value < 0.05 was considered to be statistically significant.

### Sample processing

Samples were prepared following the manufacturer’s instructions. The details were as following.

#### High-abundance protein removal

All 62 samples were depleted with top 14 high-abundant depletion spin columns (Thermo) was placed at room temperature, 10 μl (about 600 μg) of serum sample was added, and incubation was carried out for 30 min at room temperature. The supernatant was collected by centrifugation at 1,000*g* for 2 min, and the volume was about 320 μl, the solution system was 10 mM PBS, 0.15 M NaCl, 0.02% azide, pH 7.4.

#### LysC/trypsin digestion

320 μl of high-abundance protein-depleted sample was added to a 10 kD ultrafiltration tube (Millipore), 12,000*g*, centrifuged for 10 min; added 200 μl of 8 M Urea, 12,000*g*, centrifuged for 10 min, repeated once, and finally 50 μl of 8 M urea were added. Then the sample was added into a 96-well plate. 10 mM DTT solution was added and reacted at 37 °C for 30 min; IAM solution was added to a final concentration of 20 mM, and reacted at 37 °C for 30 min at room temperature in the dark for 30 min; DTT solution was added to a final concentration of 10 mM to quench the reaction. 1 μg of LysC was added to each sample, incubate at 37 °C for 2 h; 1 μg Trypsin was then added and incubated at 37 °C overnight; finally 10 μl of 10% TFA was added to terminate the reaction.

#### Desalting

Add 100 μl of methanol to SoLAμ HRP plate, 600*g*, centrifuge for 1 min; add 100 μl of 80% ACN 0.1% TFA, 1000*g*, centrifuge for 1 min; add 200 μl of 0.1% THA, 1000*g*, centrifuge for 1 min; add the sample to SoLAμ In the HRP plate, 1000*g*, centrifuge for 2 min; repeat loading the sample once; add 200 μl 0.1% THA, 1000*g*, centrifuge for 2 min to wash the column; add 100 μl 80% ACN 0.1% TFA, 1000*g*, centrifuge for 3 min, collect the elution; the elution is concentrated in the centrifuge. The peptides were dissolved to 0.5 μg/μl with 0.1% FA, and loaded with 1 μg of mass spectrometry for DIA analysis.

### LC-MS DIA proteomics analysis

DIA proteomic analysis was performed using a Thermo U3000nano RSLC nanoLC (Thermo Fisher Scientific) with Orbitrap Fusion LumosTM quadrupole-linear ion trap-electrostatic field orbitrap high resolution mass spectrometry (Thermo Fisher Scientific, USA), data acquisition software XCalibur 4.3 (Thermo Fisher Scientific). The analysis was performed following the manufacturer’s instructions, and the main parameters are as follows.

#### Liquid chromatography

Nano LC-UltiMateTM 3000 RSLC nano System; two Column Mode: Trap Column (Acclaim PepMap C18, 3 μm, 100 Å, 75 μm × 2 cm), Analytical Column (Acclaim PepMap C18, 2 μm, 100 Å, 75 μm × 25 cm); mobile phase: A: 0.1% formic acid in water; B: 0.1% formic acid in 80% acetonitrile; gradient: 3–8% B in 6 min, 8–30% B in 102 min, 30–100% B in 8 min, 100–100% B in 4 min; flow rate: 300 nL/min.

#### Mass spectrometer

Thermo Scientific Orbitrap Fusion Lumos; spray voltage: 2.1 kV; capillary temperature: 300 °C; S-lens: 50%; collision energy: 32% HCD; resolution setting: first level 60,000 m/z 200, two Level 30,000 m/z 200; Max IT: full MS 20 ms, full MS/MS 54 ms; parent ion scanning range: m/z 350–1200; product ion scanning range: start from m/z 200; number of windows: 70; isolation window: adjust the variable isolation window based on the eluted peptide m/z.

### Data processing and analysis

Spectronaut X system was used for DIA proteomics data analysis. Briefly, 62 samples and QC data were imported into Spectronaut X, targeted extracted with laboratory built serum proteomics database, and the remaining parameters were controlled by default to control FDR < 1% of peptide and protein levels. Protein intensity was calculated by Spectronaut from the average of the top3 peptide was exported. This protein intensity was imported into Perseus and Metaboanalyst for statistical analysis. Log_2_ transformation, feature filtering, missing value filling, and data normalization of raw data were done before statistical analysis. S0 0.1 and FDR *p* < 0.05 was used as the threshold value for differential proteins selection. PCA, cluster analysis, and correlation analysis were performed in Metaboanalyst. The up-regulated and down-regulated proteins were imported into the Protein Center (Thermo) for functional enrichment annotation of gene ontology (GO) and Kyoto Encyclopedia of Genes and Genomes (KEGG) with FDR *p* < 0.05. Receiver operating characteristic (ROC) curves analysis was performed using SPSS software.

### Western blotting validation

Based on the former DIA proteomics results and the identified significant protein biomarkers, Western blot assay was further performed to validate the significance of biomarkers in PCOS. Serum samples were homogenized in RIPA buffer containing protease inhibitor and the protein concentration was measured using a BCA method. 10% sodium dodecyl sulphate–polyacrylamide gel electrophoresis (SDS-PAGE) was used to separate the proteins, then the proteins were electrotransferred onto the nitrocellulose (NC) membranes. After blocking with non-fat mike for 1 h and washed with Tris-buffered saline containing Tween 20 (TBST), the membranes were probed with the primary antibodies against Histone H4 (H4, Affinity, dilution 1:2000), Histone H2A (H2A, Affinity, dilution 1:1000), Trem-like transcript 1 protein (TLT-1, R&D systems, 0.1 µg/mL), Peroxiredoxin-1 (PRDX1, Affinity, dilution 1:2000), Band 3 anion transport protein (SLC4A1, Affinity, dilution 1:2000), Transferrin (Affinity, dilution 1:2000) at 4 °C over-night. And subsequently followed by incubated with horseradish peroxidase-conjugated secondary antibodies for 2 h at room temperature. Transferrin was detected as an internal reference protein [12]. Protein bands were visualized by enhanced chemiluminescence reagents and the protein intensity was quantified using Image-J software. Data was expressed as mean ± standard deviation, group comparisons were processed using the two-tailed Student’s *t*-test, *p* < 0.05 was considered as statistical significance.

## Results

### Clinical characteristics and biochemical data of the study subjects

The clinical characteristics and biochemical data of the study subjects were collected and analyzed, and the relative results were presented in Table 1. In this study, the study subjects included 31 healthy women as controls and 31 PCOS women. There are no statistically differences for the age and BMI between the two groups (*p* value > 0.05). For PCOS related biochemical data, the levels of fasting glucose, LH, T, TG, LDL-c and LH/FSH ratio were significantly higher in PCOS patients than those in the controls, the levels of PRL, HDL-c were significantly lower in PCOS patients than those in the controls (*p* value < 0.05).

**Table 1:** Clinical characteristic of the study subjects

	Control (n = 31)	PCOS (n = 31)	*p* value
Age [years]	24.52 ± 2.31	24.20 ± 4.49	0.750
BMI [kg/m^2^]	20.48 ± 2.67	22.27 ± 3.56	0.081
Fasting glucose [mmol/L]	4.68 ± 0.42	5.25 ± 1.20	0.026 < 0.05
FSH [IU/L]	5.15 ± 1.32	5.59 ± 2.86	0.440
LH [IU/L]	5.85 ± 2.74	9.82 ± 8.57	0.017 < 0.05
LH/FSH	1.14 ± 0.50	1.73 ± 0.97	0.003 < 0.01
PRL [mIU/L]	466.83 ± 231.05	309.64 ± 158.59	0.003 < 0.01
E2 [pmol/L]	209.95 ± 127.20	220.25 ± 246.80	0.837
T [nmol/L]	1.12 ± 0.40	1.65 ± 0.67	< 0.001
P [nmol/L]	0.93 ± 0.36	1.05 ± 0.80	0.451
TC [mmol/L]	4.44 ± 0.63	4.65 ± 0.76	0.306
TG [mmol/L]	0.75 ± 0.29	1.13 ± 0.51	0.002 < 0.05
HDL-c [mmol/L]	2.23 ± 0.52	1.46 ± 0.41	< 0.001
LDL-c [mmol/L]	1.69 ± 0.29	2.54 ± 0.62	< 0.001

### Quality control of the proteomics analysis

**Fig. 1:**
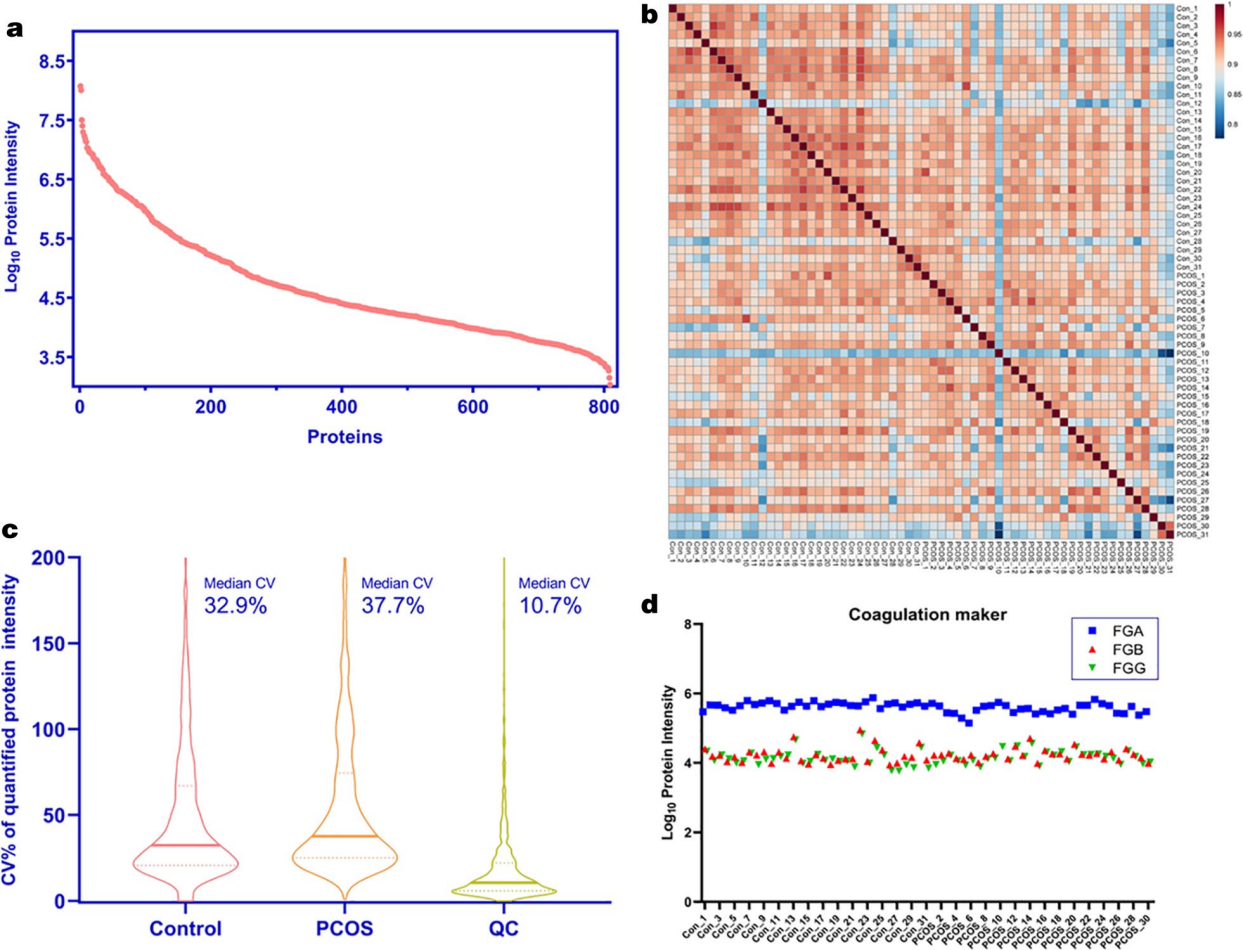
Quality control analysis of the proteomics analysis. **a** Dynamic range of 809 quantified proteins. X-protein numbers, Y-Log10 protein intensities. **b** Box plot of CV (%) in each sample group, PCOS, Control and QC group. Median CV were also indicated. **c** Person correlation in 62 samples. **d** Protein Intensity of FGA, FGB and FGG in 62 serum samples. No of samples much lower abundance of FGA, FGB and FGG indicated coagulation during serum collection. CV: coefficient of variation; QC: quality control; FGA: Fibrinogen alpha chain; FGB: Fibrinogen beta chain; FGG: Fibrinogen gamma chain

The intensity of all identified proteins showed that the abundance of these proteins spans largely, covering 5 orders of magnitude, suggesting that the instrument is more sensitive (Fig. 1a). Coefficient of variation (CV) of the control group and PCOS group samples were 37.7% and 32.9%, respectively. Pearson correlation analysis of the protein intensity showed that the correlation index was among 0.8–1, suggested that the experimental procedures are reproducible (Fig. 1b, c). Blood coagulation is a interference factor for serum proteomics analysis, the decrease of fibrinogen indicates the coagulation event. We detected the serum levels of fibrinogen alpha chain (FGA), fibrinogen beta chain (FGB), fibrinogen gamma chain (FGG), the results showed that there are no significant decreases of FGA, FGB and FGG in all samples, suggested there is no coagulation event in our tested samples (Fig. 1d).

### Principal component analysis (PCA)

**Fig. 2:**
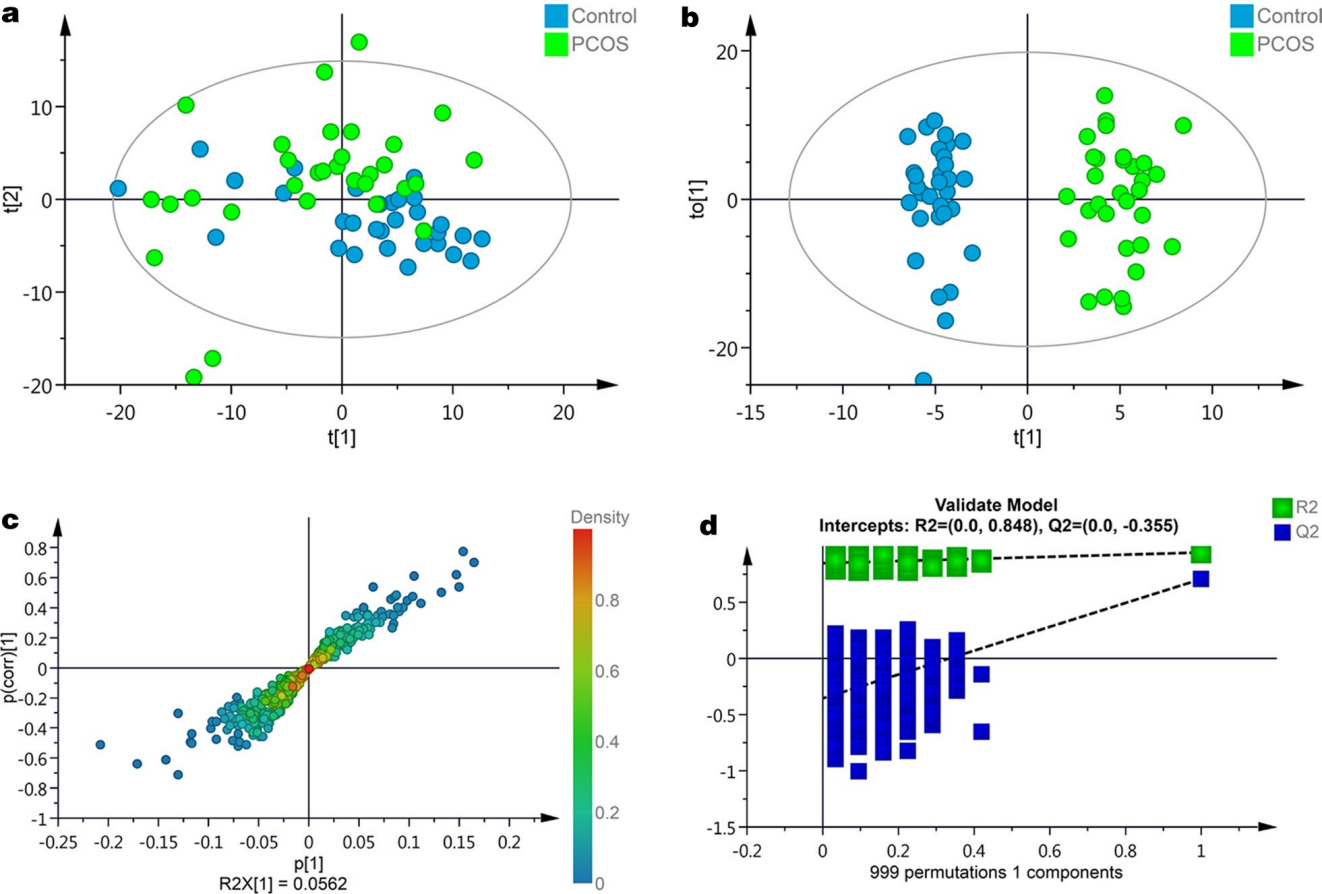
Principal component analysis of proteome profiles. **a** Score plot of PCA analysis to overview classification trend of proteome profiles of PCOS and Control groups. Model parameter: R2X = 0.46 (cumulative variance proportion of 8 principal components). **b** Score plot of OPLS-DA modeling to maximize inter-group differentiation of proteomic data between PCOS and Control groups. Model parameter: 1 predictive component + 2 orthogonal components, R^2^Y = 0.94, Q^2^ = 0.71. **c** S-plot of OPLS-DA modeling using DIA proteomics quantitative data. **d** 999 times permutation test of OPLS-DA modeling

There were 550 proteins were identified and filtered from the dataset. The PCA and OPLS-DA were performed based on the quantitative data of these proteins to determine the principal axes on protein abundance variations in PCOS cases and controls. The PCA (Fig. 2a) showed moderate separation of the serum proteins between the PCOS and control group at t(2) axis, but with overlaps at t(1) axis. Further OPLS-DA result (Fig. 2b, c) showed complete separation of the serum proteins between the PCOS and control group. The quality parameters of the model were: R^2^Y = 0.94; Q^2^ = 0.71. The OPLS-DA model was also verified by 999 times permutation test (Fig. 2d).

### Proteomics in PCOS patients and controls

**Fig. 3:**
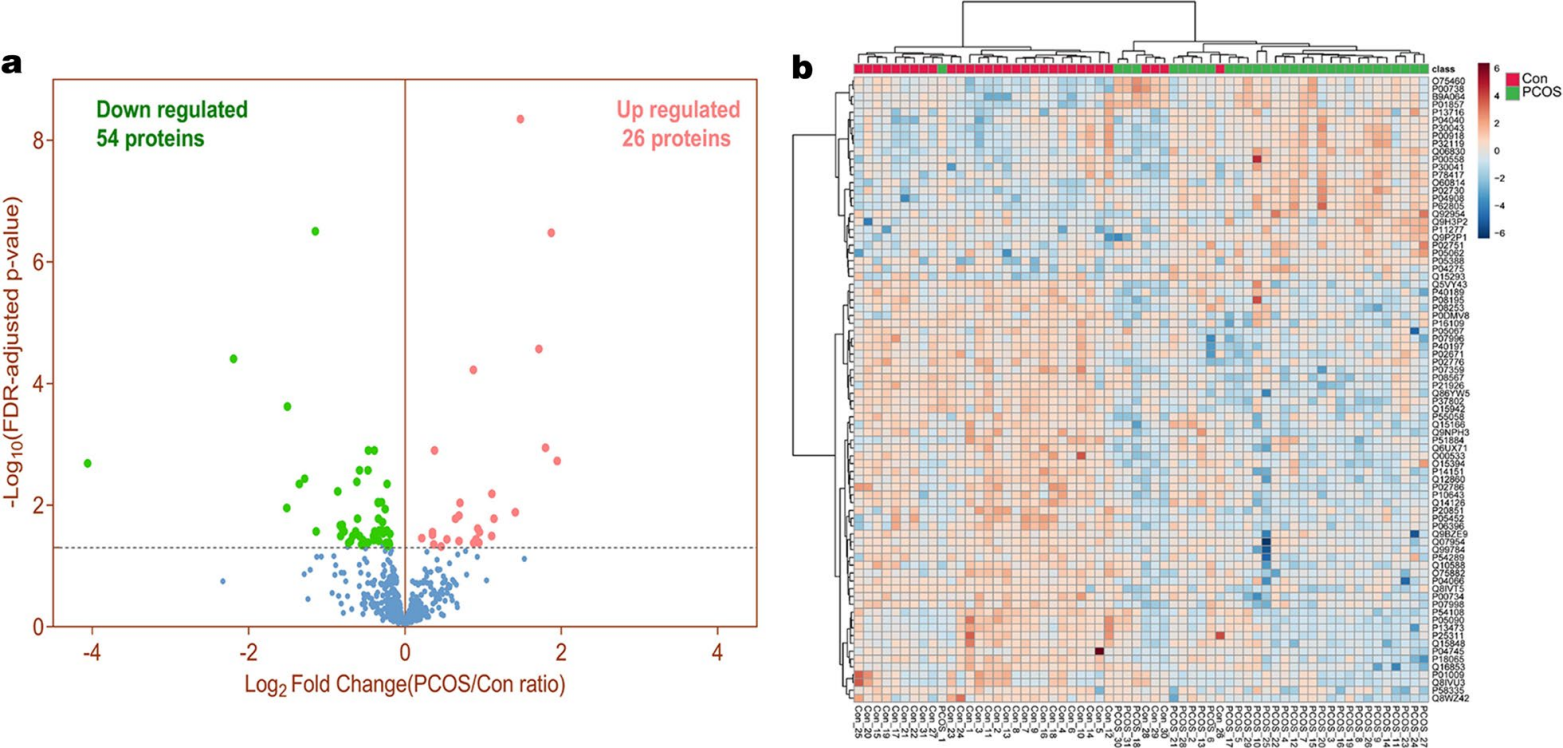
Identification of 26 up-regulated and 54 down regulated proteins. **a** Volcano plot of quantitative DIA proteome data visualizing A/Con. Proteins with Q < 0.05 as significant proteins were red highlight. Number of upregulated and down-regulated proteins were indicated in each volcano plot. **b** Heatmap and clustering analysis result of differential proteins in *p* value of significance level *p* value < 0.05 after FDR adjustment

A total of 80 proteins were significantly differentially abundant in PCOS patients compared to the healthy controls from the criterion *p* value after Benjamin-Hocheberg FDR adjustment p value was less than 0.05. The detailed information of these proteins were presented in Table 2. Among these proteins, 56 were down-regulated, 24 were up-regulated (Fig. 3a). Heatmap (Fig. 3b) also showed the expression intensity of these proteins, and these proteins were clustered significantly in the samples between the PCOS and control groups.

**Table 2:** List of the 80 differential expressed proteins in PCOS patients plasma

No	Protein accessions	Proteins	Proteins	FDR adj *p* value PCOS_Con	PCOS/Con ratio	− Log10 (FDR adj *p* value PCOS/Con)
1	O60814	H2B	Histone H2B	1.87E−03	3.852	2.729
2	P04908	H2A	Histone H2A	3.29E−07	3.658	6.482
3	P11277	SPTB	Spectrin beta chain, erythrocytic	1.13E−03	3.475	2.945
4	P02730	SLC4A1	Band 3 anion transport protein	2.70E−05	3.279	4.569
5	P62805	H4	Histone H4	4.47E−09	2.784	8.350
6	P05388	RPLP0	60S acidic ribosomal protein P0	1.30E−02	2.660	1.886
7	O75460	ERN1	Serine/threonine-protein kinase/endoribonuclease IRE1	1.66E−02	2.201	1.781
8	P30041	PRDX6	Peroxiredoxin-6	6.48E−03	2.160	2.189
9	B9A064	IGLL5	Immunoglobulin lambda-like polypeptide 5	3.17E−02	2.154	1.498
10	P04040	CAT	Catalase	2.76E−02	1.934	1.559
11	P00738	HP	Haptoglobin	4.10E−02	1.926	1.387
12	P13716	ALAD	Delta-aminolevulinic acid dehydratase	2.41E−02	1.903	1.618
13	Q9P2P1	NYNRIN	Protein NYNRIN	3.62E−02	1.875	1.441
14	P01857	IGHG1	Ig gamma-1 chain C region	4.16E−02	1.835	1.380
15	Q06830	PRDX1	Peroxiredoxin-1	5.92E−05	1.832	4.228
16	P32119	PRDX2	Peroxiredoxin-2	9.11E−03	1.629	2.040
17	P05062	ALDOB	Fructose-bisphosphate aldolase B	3.85E−02	1.614	1.415
18	P30043	BLVRB	Flavin reductase (NADPH)	1.48E−02	1.612	1.830
19	P00918	CA2	Carbonic anhydrase 2	1.66E−02	1.563	1.781
20	Q15293	RCN1	Reticulocalbin-1	3.62E−02	1.450	1.441
21	P00558	PGK1	Phosphoglycerate kinase 1	4.75E−02	1.376	1.323
22	P02751	FN1	Fibronectin	1.25E−03	1.298	2.904
23	Q92954	PRG4	Proteoglycan 4	4.37E−02	1.289	1.359
24	P78417	GSTO1	Glutathione S-transferase omega-1	3.04E−02	1.276	1.517
25	P04275	VWF	von Willebrand factor	2.76E−02	1.275	1.559
26	Q9H3P2	NELFA	Negative elongation factor A	3.45E−02	1.160	1.462
27	Q6UX71	PLXDC2	Plexin domain-containing protein 2	2.94E−02	0.876	1.531
28	P08195	SLC3A2	4F2 cell-surface antigen heavy chain	4.39E−02	0.872	1.357
29	P51884	LUM	Lumican	4.10E−02	0.866	1.388
30	O75882	ATRN	Attractin	4.08E−02	0.864	1.390
31	P20851	C4BPB	C4b-binding protein beta chain	4.66E−02	0.857	1.331
32	P00734	F2	Prothrombin	4.45E−03	0.852	2.351
33	P14151	SELL	L-selectin	2.59E−02	0.851	1.586
34	P06396	GSN	Gelsolin	4.16E−02	0.844	1.380
35	O00533	CHL1	Neural cell adhesion molecule L1-like protein	1.15E−02	0.838	1.938
36	P55058	PLTP	Phospholipid transfer protein	2.88E−02	0.824	1.541
37	P07359	GP1BA	Platelet glycoprotein Ib alpha chain	1.89E−02	0.819	1.723
38	P08253	MMP2	72 kDa type IV collagenase	2.59E−02	0.816	1.586
39	Q14126	DSG2	Desmoglein-2	1.87E−02	0.814	1.728
40	Q9NPH3	IL1RAP	Interleukin-1 receptor accessory protein	8.88E−03	0.811	2.051
41	P07998	RNASE1	Ribonuclease pancreatic	2.59E−02	0.811	1.586
42	P02776	PF4	Platelet factor 4	2.70E−02	0.806	1.569
43	P07996	THBS1	Thrombospondin-1	2.59E−02	0.797	1.586
44	Q07954	LRP1	Prolow-density lipoprotein receptor-related protein 1	3.81E−02	0.793	1.419
45	Q15166	PON3	Serum paraoxonase/lactonase 3	1.66E−02	0.790	1.781
46	P05452	CLEC3B	Tetranectin	9.11E−03	0.789	2.040
47	P10643	C7	Complement component C7	8.88E−03	0.788	2.051
48	P54289	CACNA2D1	Voltage-dependent calcium channel subunit alpha-2/delta-1	3.49E−02	0.780	1.457
49	P05067	APP	Amyloid beta A4 protein	2.70E−02	0.764	1.569
50	Q12860	CNTN1	Contactin-1	1.25E−03	0.762	2.904
51	Q99784	OLFM1	Noelin	3.71E−02	0.758	1.431
52	P40189	IL6ST	Interleukin-6 receptor subunit beta	3.17E−02	0.756	1.498
53	P02671	FGA	Fibrinogen alpha chain	1.25E−03	0.723	2.904
54	P40197	GP5	Platelet glycoprotein V	2.65E−03	0.720	2.576
55	Q9BZE9	ASPSCR1	Tether containing UBX domain for GLUT4	4.13E−02	0.720	1.384
56	Q10588	BST1	ADP-ribosyl cyclase/cyclic ADP-ribose hydrolase 2	3.71E−02	0.688	1.431
57	P13473	LAMP2	Lysosome-associated membrane glycoprotein 2	4.49E−02	0.682	1.348
58	P04066	FUCA1	Tissue alpha-L-fucosidase	4.37E−02	0.680	1.359
59	Q8IVU3	HERC6	Probable E3 ubiquitin-protein ligase HERC6	3.17E−02	0.668	1.498
60	P02786	TFRC	Transferrin receptor protein 1	2.66E−03	0.667	2.575
61	P25311	AZGP1	Zinc-alpha-2-glycoprotein	1.66E−02	0.656	1.781
62	Q8WZ42	TTN	Titin	4.10E−03	0.653	2.388
63	P01009	SERPINA1	Alpha-1-antitrypsin	2.70E−02	0.645	1.569
64	P05090	APOD	Apolipoprotein D	3.17E−02	0.629	1.498
65	P16109	SELP	P-selectin	3.88E−02	0.618	1.412
66	Q15848	ADIPOQ	Adiponectin	4.16E−02	0.607	1.380
67	P04745	AMY1A	Alpha-amylase 1	2.70E−02	0.582	1.569
68	Q16853	AOC3	Membrane primary amine oxidase	2.45E−02	0.575	1.611
69	P54108	CRISP3	Cysteine-rich secretory protein 3	2.11E−02	0.573	1.676
70	P58335	ANTXR2	Anthrax toxin receptor 2	3.17E−02	0.565	1.498
71	Q5VY43	PEAR1	Platelet endothelial aggregation receptor 1	2.13E−02	0.563	1.671
72	P18065	IGFBP2	Insulin-like growth factor-binding protein 2	5.87E−03	0.551	2.231
73	P0DMV8	HSPA1A	Heat shock 70 kDa protein 1A	2.70E−02	0.455	1.569
74	Q86YW5	TLT-1	Trem-like transcript 1 protein	3.11E−07	0.452	6.507
75	P21926	CD9	CD9 antigen	3.64E−03	0.411	2.438
76	P08567	PLEK	Pleckstrin	4.45E−03	0.392	2.351
77	P37802	TAGLN2	Transgelin-2	2.37E−04	0.352	3.626
78	O15394	NCAM2	Neural cell adhesion molecule 2	1.10E−02	0.350	1.957
79	Q15942	ZYX	Zyxin	3.91E−05	0.219	4.408
80	Q8IVT5	KSR1	Kinase suppressor of Ras 1	2.04E−03	0.060	2.691

### Bioinformatics analysis of the proteomic results

**Fig. 4:**
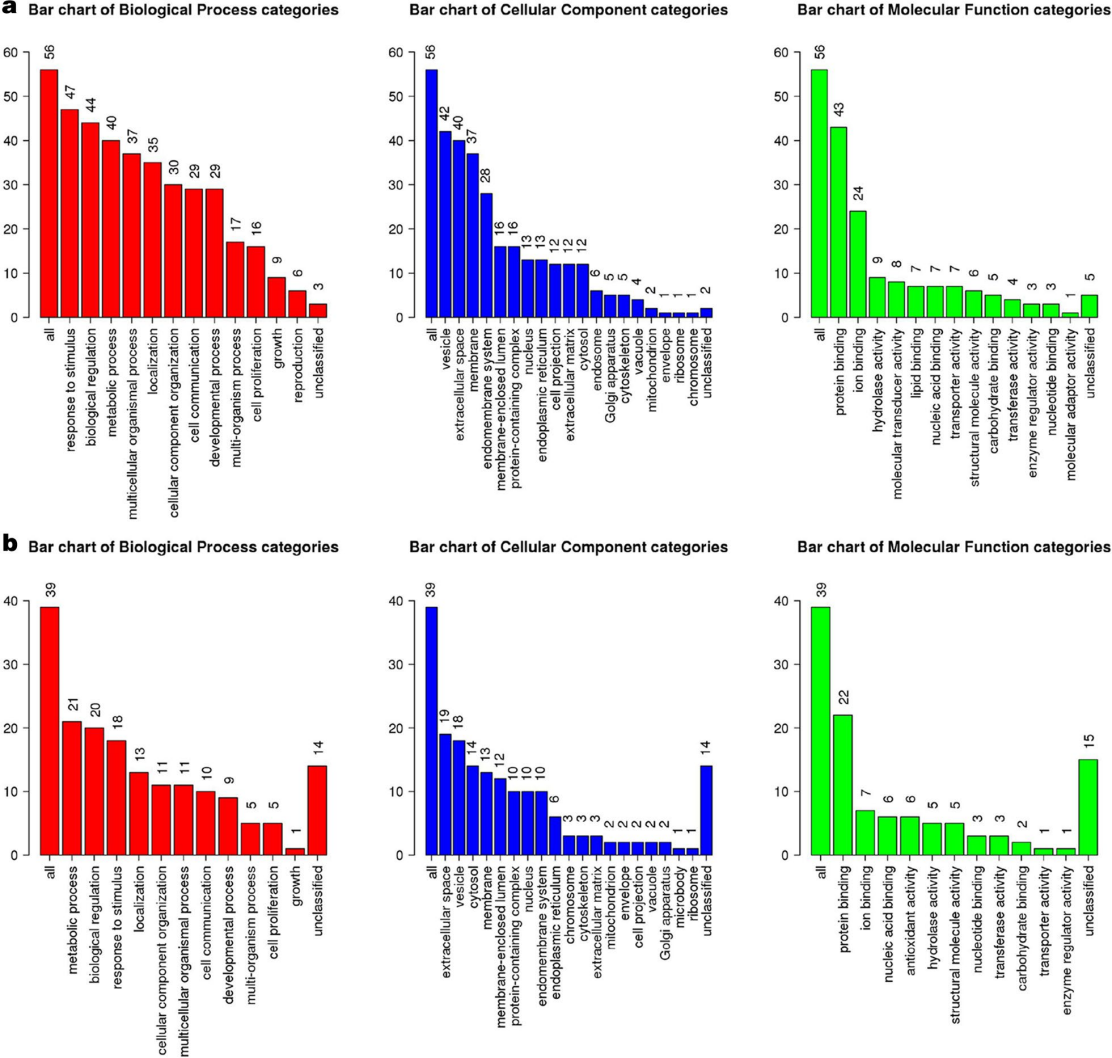
Volcano plot of GO (GOBP, GOCC and GOMF) and KEGG enrichment result using over-representation method. Left: 54 DOWN-regulated proteins in PCOS group compared to sample Control group; Right: 26 Up-regulated proteins in PCOS group compared to Control group. Those genesets with FDR adjusted *p* value < 0.05 were labeled

**Fig. 5:**
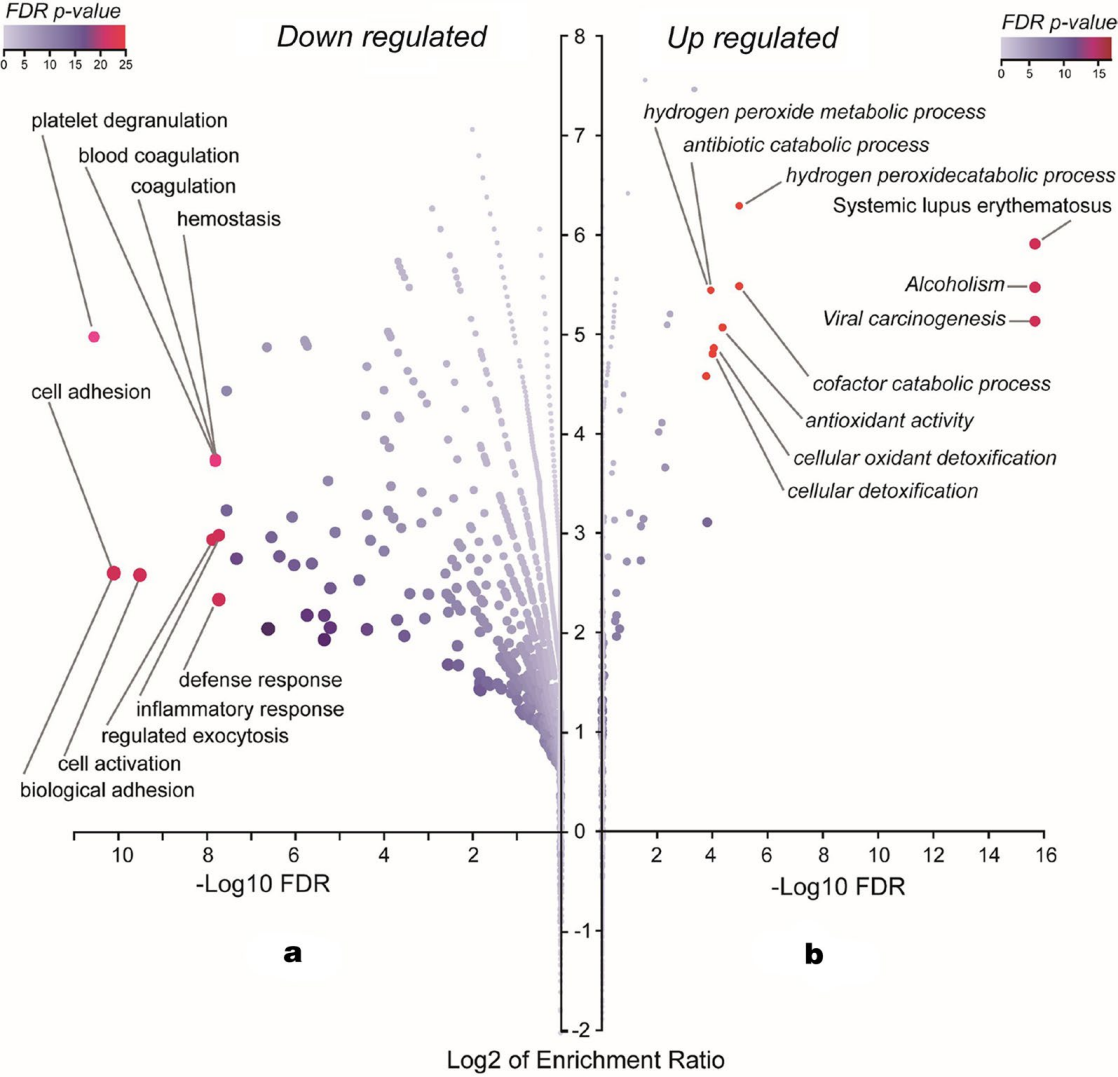
Gene ontology analysis of the proteins. **a** GOBP, GOCC and GOMP enrichment results of 54 downregulated proteins. **b** GOBP, GOCC and GOMP enrichment results of 26 upregulated proteins. Y axis: protein numbers. GOBP: gene ontology biological process; GOCC: gene ontology cellular component; GOMP: gene ontology molecular function

The GO terms of these 56 down-regulated and 24 up-regulated proteins enriched were shown in Fig. 4. GO terms were divided into three categories, biological process (BP), cellular component (CC), and molecular function (MF). GO analysis of the downregulated proteins showed that the enriched BP terms include response to stimulus, biological regulation, metabolic process, cell proliferation, and especially reproduction term. The GO terms that upregulated proteins enriched were similar to those downregulated proteins. Volcano plot (Fig. 5) of GO and KEGG enrichment results showed that 54 downregulated proteins were enriched in terms including platelet degranulation, cell adhesion, biological adhesion, cell activation, regulated exocytosis, blood coagulation, coagulation, hemostasis, defense response and inflammatory response. 26 upregulated proteins were enriched in terms including viral carcinogenesis, alcoholism, systemic lupus erythematosus, cofactor catabolic process, hydrogen peroxide catabolic process, antioxidant activity, cellular oxidant detoxification, cellular detoxification, antibiotic catabolic process and hydrogen peroxide metabolic process.

### ROC curves analysis

**Fig. 6:**
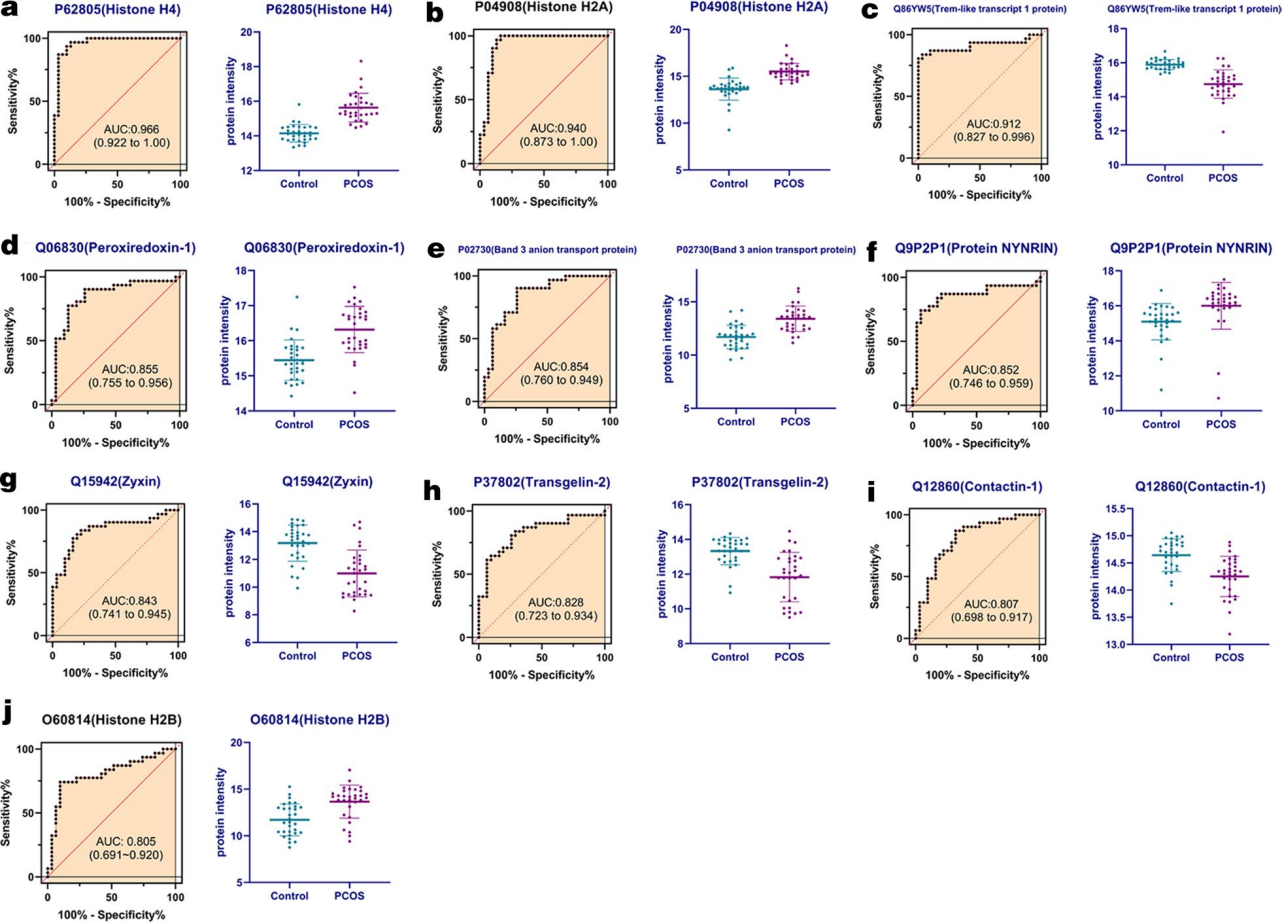
ROC-curve analysis of top 10 proteins ranked AUC values

In order to explore the diagnoses potential of these 80 proteins in PCOS cases, all the 80 proteins were further introduced for ROC curves analysis. And top 10 proteins with the higher area under curve (AUC) were presented in Fig. 6 and Table 3, including H4, H2A, TLT-1, PRDX1, SLC4A1, Protein NYNRIN (NYNRIN), Zyxin (ZYX), Transgelin-2 (TAGLN2), Contactin-1 (CNTN1), Histone H2B (H2B). It could be observed that the AUC of H4, H2A, TLT-1 were all over than 0.9 (AUC = 0.966, 0.940, 0.912, respectively), and they were all differentially abundant in PCOS and control subjects significantly, indicated promising diagnosis values of these proteins.

**Table 3:** Top 10 proteins ranked based on area under ROC curve

Name	Protein	AUC	FC(PCOS/Con)	*p* value
P62805	Histone H4	0.966	2.79	4.47E−09
P04908	Histone H2A	0.940	3.66	3.29E−07
Q86YW5	Trem-like transcript 1 protein	0.912	0.45	3.11E−07
Q06830	Peroxiredoxin-1	0.855	1.83	5.92E−05
P02730	Band 3 anion transport protein	0.854	3.27	2.70E−05
Q9P2P1	Protein NYNRIN	0.852	1.88	3.62E−02
Q15942	Zyxin	0.843	0.22	3.91E−05
P37802	Transgelin-2	0.828	0.35	2.37E−04
Q12860	Contactin-1	0.807	0.76	1.25E−03
O60814	Histone H2B	0.805	3.86	1.87E−03

ROC, receiver operating characteristic; AUC, area under curve; FC, fold change

### Validation the expression of significant proteins

**Fig. 7:**
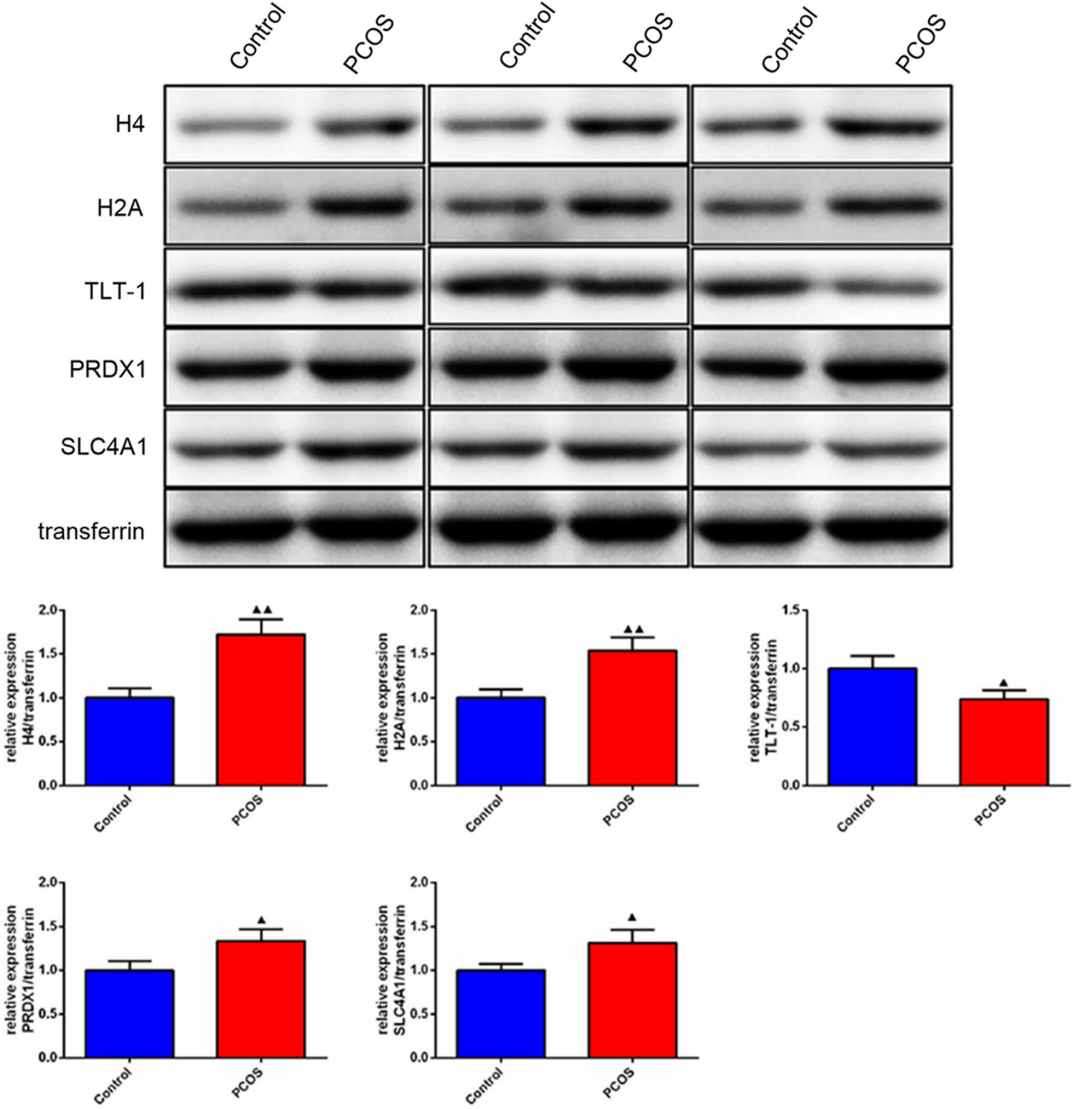
Western blot analysis of top five significant proteins (H4, H2A, TLT-1, PRDX1, SLC4A1). n = 3, compared to the control group, ^▲^*p* < 0.05, ^▲▲^*p* < 0.01

Based on the former DIA proteomics results and the identified significant biomarkers, we chose the expression of top five significant proteins (H4, H2A, TLT-1, PRDX1, SLC4A1) from the ROC curves results for further validation using Western blot analysis. As shown in Fig. 7, the expression of H4, H2A, PRDX1, SLC4A1 in PCOS group were significantly increased comparing to the control group, but the expression of TLT-1 was significantly decreased in PCOS group comparing to the control group. More importantly, tendency of these detected proteins between the PCOS and control groups were consisted with the DIA proteomics results.

## Discussion

Despite the great potential of -omic sciences in elucidating biological processes and monitoring disease progression, comprehensive proteomic analysis on PCOS patients has not been investigate fully to date [8, 13]. In this study, by quantitative DIA proteomics, we aimed to investigate whether proteomics change in PCOS women serum samples compared to the healthy controls, and the results evidenced the serum proteomic profile alterations in PCOS female. As a result, there were 80 proteins significantly differentially expressed between PCOS patients and controls, including 54 downregulated and 26 upregulated proteins. GO and KEGG analysis showed that downregulated proteins were enriched in platelet degranulation, cell adhesion, cell activation, blood coagulation, hemostasis, defense response and inflammatory response terms; upregulated proteins were enriched in cofactor catabolic process, hydrogen peroxide catabolic process, antioxidant activity, cellular oxidant detoxification, cellular detoxification, antibiotic catabolic process and hydrogen peroxide metabolic process. ROC curves analysis showed that the AUC of H4, H2A, TLT-1 were all over than 0.9, indicated promising diagnosis values of these proteins.

Serum proteomics results showed that there were 80 proteins differentially expressed between PCOS patients and controls significantly. Further GO and KEGG enrichment analysis indicated that the downregulated proteins were enriched in platelet degranulation, blood coagulation and inflammatory response. Reproductive disorders, such as PCOS, are often accompanied by platelet dysfunctions and thereby induced inflammation [14]. In patients with PCOS, coagulation and fibrinolysis parameters are usually evaluated. Platelet involves in granulosa cell corpus luteum formation and angiogenesis in ovary, and abnormal follicular development in PCOS is partly contributed by ovarian angiogenesis dysregulation. Aye et al. examined the effect of hypertriglyceridemia on IR and platelet function in young women with PCOS, and reported that acute hypertriglyceridemia induced IR, increased platelet activation both in control and PCOS groups [15]. PCOS patients also have risk to induce prothrombotic state, and platelet dysfunction might responsible for it. This study showed that changed proteins were enriched in platelet degranulation and blood coagulation in PCOS group, which indicated that these protein changes might contributed to the platelet dysfunction, and also associated with IR and inflammation. GO results also showed that these changed proteins were also enriched in reproduction biological process, which indicated that the proteomics changes in PCOS cases were directly associated with the PCOS pathogenesis and induced infertility. In addition, PCOS is also a low-grade chronic inflammation disease, continuous releasing of inflammatory mediators could perpetuate the inflammatory condition in women with PCOS [16, 17]. Proteomics changes can also impact on inflammatory response and reveal the presence of inflammation in disease [18]. For the identified proteins that enriched in inflammatory response in this analysis, some proteins were related to blood coagulation, including thrombin (also called coagulation factor II), platelet factor 4, thrombospondin 1. Some publications also reported the associations of them with inflammation of PCOS [19–21]. Platelets are inflammatory anuclear cells, blood coagulation is an intrinsic pathway for proinflammation, blood coagulation factors also are important inflammatory mediators than just promoting or inhibiting blood coagulation [22–24]. The activation of coagulation factors could cause a proinflammatory response and initiate coagulation and downstream cellular signaling pathways.

Our ROC curves results showed that the AUC of H4, H2A, TLT-1 were all over than 0.9, indicated a significant role of these three proteins in differentiating PCOS from controls. Among these three proteins, proteomics analysis showed that H4 and H2A was upregulated in PCOD patients with a PCOS/control ratio of 2.79 and 3.66, respectively. Histone is one of the critical components of chromatin, the amino acid residues at its N-terminus can be covalently modified, thus change the chromatin conformation and induce transcription or gene silencing [25]. This modification mainly including acetylation/deacetylation, methylation/demethylation, ubiquitination/deubiquitination, phosphorylation, sumo, biotin, etc. Excepting for gene expression controlling, histone modification also participates in cell division, cell apoptosis and memory formation by recruiting protein complex and affecting downstream proteins, and also has the impact on immune system and inflammatory reaction [26]. Histone subunits include H2A, H2B, H3 and H4, the histone modifications aforementioned could all be occurred and thus exhibit multiple functions. But previous studies about H4 or H2A in PCOS is rare. Monteiro’s study reported that in endometriosis patients, lesions had significantly lower levels of Histone H3K9ac and Histone H4K16 acetylation compared to eutopic endometrium from controls, and comparing to the control endometrium, the hypoacetylation of Histone H3/H4 within promoter regions of candidate genes known to be downregulated in endometriosis lesions, while the stereoidogenic factor 1 promoter region was enriched for acetylated H3 and H4 in lesions versus control tissues, correlating with its reported high expression in lesions [27]. Neonatal exposure to diethylstilbestrol (DES) can cause permanent alterations in female reproductive tract gene expression, infertility, and uterine cancer in mice, after DES treatment, three histone modifications associated with active transcription, including Histone H4 lysine 5 acetylation (H4K5ac), which were found to enriched at specific lactoferrin (Ltf) promoter regions in uterine [28]. This suggested that the alteration expression of multiple chromatin-modifying proteins and epigenetic marks might lead to altered reproductive function and increased cancer risk.

TLT-1 is another protein with AUC over than 0.9 in ROC analysis. But different from the aforementioned two proteins, our proteomics analysis showed that TLT-1 was downregulated in PCOD patients with a PCOS/control ratio of 0.452. TLT-1 belongs to a kind of triggering receptors expressed on myeloid cells that play important roles in innate and adaptive immune responses, platelet aggregation, inflammation and insulted bleeding [29]. It mediates blood coagulation via binding fibrinogen. Based on the critical role in immune, inflammation and platelets, previous studies of TLT-1 are mainly focused on these associated diseases. In patients with systemic lupus erythematosus, the soluble TLT-1 levels were significantly lower than healthy individuals [30]. In a model of acute lung injury, results showed that infusion of sTLT-1 restored normal fibrinogen deposition and alleviated pulmonary hemorrhage by 40% and tissue damage by 25% [31]. In thrombocytopenia and platelet function defect, the content of TLT-1 was reduced, recombinant soluble TLT-1 could potentiate fibrinogen binding to patient platelets, and TLT-1 was found to be positively regulated by RUNX1 [32]. Derive et al. reported that TLT-1 is a potent endogenous regulator of sepsis-associated inflammation via suppressing leukocyte activation and modulating platelet-neutrophil crosstalk [33]. In this study, the expression of TLT-1 was also downregulated, and AUC of ROC curves analysis was 0.912. In this cases, we suspected that the changed expression of TLT-1 in PCOS might involved in the mediation of chronic inflammation and blood coagulation, thus impacting on the pathogenesis of PCOS.

In conclusion, this study enrolled 31 PCOS patients and 31 matched healthy control participators, by quantitative DIA proteomics analysis, we provided evidence of serum proteomic profile alterations in female with PCOS. 80 proteins were significantly differentially expressed between PCOS patients and controls, including 54 downregulated and 26 upregulated proteins. GO and KEGG analysis showed that downregulated proteins were enriched in platelet degranulation, cell adhesion, cell activation, blood coagulation, hemostasis, defense response and inflammatory response terms; upregulated proteins were enriched in cofactor catabolic process, hydrogen peroxide catabolic process, antioxidant activity, cellular oxidant detoxification, cellular detoxification, antibiotic catabolic process and hydrogen peroxide metabolic process. ROC curves analysis showed that the AUC of H4, H2A, TLT-1 were all over than 0.9, indicated promising diagnosis values of these proteins in PCOS. Western blotting also validated that H4, H2A, TLT-1, PRDX1, SLC4A1 were differently expressed in PCOS and control groups significantly. Future studies comparing systemic expression and exact role of these candidate biomarkers in PCOS are essential for confirmation of this hypothesis.

## Data Availability

The datasets supporting the conclusions of this article are included within the article.
